# Possible Fruit Protein Effects on Primate Communities in Madagascar and the Neotropics

**DOI:** 10.1371/journal.pone.0008253

**Published:** 2009-12-16

**Authors:** Jörg U. Ganzhorn, Summer Arrigo-Nelson, Sue Boinski, An Bollen, Valentina Carrai, Abigail Derby, Giuseppe Donati, Andreas Koenig, Martin Kowalewski, Petra Lahann, Ivan Norscia, Sandra Y. Polowinsky, Christoph Schwitzer, Pablo R. Stevenson, Mauricio G. Talebi, Chia Tan, Erin R. Vogel, Patricia C. Wright

**Affiliations:** 1 Biozentrum Grindel, University of Hamburg, Hamburg, Germany; 2 Interdepartmental Doctoral Program in Anthropological Sciences, Ecology and Evolution, Stony Brook University, Stony Brook, New York, United States of America; 3 Department of Anthropology, University of Florida, Gainesville, Florida, United States of America; 4 University of Antwerp, Department of Biology, Wilrijk, Belgium and Center for Research and Conservation, Royal Zoological Society of Antwerp, Antwerp, Belgium; 5 Department of Biology, University of Pisa, Pisa, Italy; 6 Department of Anthropology, Stony Brook University, Stony Brook, New York, United States of America; 7 Forschungsinstitut für die Biologie landwirtschaftlicher Nutztiere, Dummerstorf, Germany; 8 Bristol Conservation and Science Foundation, Bristol Zoo Gardens, Clifton, Bristol, United Kingdom; 9 Department of Biological Anthropology, University of Cambridge, Cambridge, United Kingdom; Stanford University, United States of America

## Abstract

**Background:**

The ecological factors contributing to the evolution of tropical vertebrate communities are still poorly understood. Primate communities of the tropical Americas have fewer folivorous but more frugivorous genera than tropical regions of the Old World and especially many more frugivorous genera than Madagascar. Reasons for this phenomenon are largely unexplored. We developed the hypothesis that Neotropical fruits have higher protein concentrations than fruits from Madagascar and that the higher representation of frugivorous genera in the Neotropics is linked to high protein concentrations in fruits. Low fruit protein concentrations in Madagascar would restrict the evolution of frugivores in Malagasy communities.

**Methodology/Principal Findings:**

We reviewed the literature for nitrogen concentrations in fruits from the Neotropics and from Madagascar, and analyzed fruits from an additional six sites in the Neotropics and six sites in Madagascar. Fruits from the Neotropical sites contain significantly more nitrogen than fruits from the Madagascar sites. Nitrogen concentrations in New World fruits are above the concentrations to satisfy nitrogen requirements of primates, while they are at the lower end or below the concentrations to cover primate protein needs in Madagascar.

**Conclusions/Significance:**

Fruits at most sites in the Neotropics contain enough protein to satisfy the protein needs of primates. Thus, selection pressure to develop new adaptations for foods that are difficult to digest (such as leaves) may have been lower in the Neotropics than in Madagascar. The low nitrogen concentrations in fruits from Madagascar may contribute to the almost complete absence of frugivorous primate species on this island.

## Introduction

Primate communities of Madagascar are known for the paucity of frugivorous species. In contrast, the high representation of frugivores but under-representation of truly folivorous vertebrates in the Neotropics has been a long-standing enigma in ecology [1]–[3]. Neotropical primate communities (often used as proxy for mammal communities in general [1], [3], [4]), contain more frugivorous genera and species when compared to the Old World primate radiations of Africa/Asia, and these in turn have more frugivores than primate communities of Madagascar [5], [6]. Explanations for the different numbers of frugivores and folivores include phenological patterns of food resources and plant species diversity. First, it has been postulated for the Neotropics, that young leaves are rare at the time of year when fruit abundance is low. This makes it unlikely that species in the Americas can fall back on young leaves during times of fruit shortage [2] and does not favor the evolution of folivores. Second, food plant diversity, and in particular the regional species richness of figs as keystone fruit trees during times of food shortage, has been linked to the diversity of frugivores [7], [8]. Madagascar has very few species of figs and fruit production is erratic due to high climatic stochasticity [9]–[12]. Thus, both factors may contribute to the paucity of frugivores in Madagascar though the generalization of both hypotheses has been questioned and modified by analyses of extended datasets [13]–[15].

Here, we propose a supplementary hypothesis to explain the higher representation of frugivorous taxa in New World tropical communities compared to Madagascar. Protein, measured as nitrogen concentration, is assumed to be a limiting factor in many communities [16]. For primates, the biomass but not the diversity of folivores has been linked to the ratio of nitrogen to fiber in leaves of a given forest [17]–[19], indicating a strong effect of protein availability on folivorous primates, though the biologically most appropriate measures for protein availability in leaves are still being developed further [20], [21].

Our hypothesis is based on a somewhat different argument. The key assumption for the evolution of primate diversity postulates that primates evolved under the constraints of protein availability [22], [23]. According to this hypothesis, fruits, - although central food resources because they are easy to digest and protein digestion is not hindered by secondary plant components to the extent seen in leaves - , are not supposed to contain enough protein to satisfy the requirements of primates. Therefore, species were forced to add leaves (if large-bodied) or insects (if small-bodied) to satisfy their nitrogen needs [22]–[24], and supplement their diets with alternative food resources during times of fruit shortage [25].

There is no doubt that unusual environmental conditions and food shortage can cause famine and death in primates, and thus, lean seasons require special adaptations for survival [26]–[28]. But, large-bodied species store nutrients when they are available. This type of “capital breeding” seems to be favored when maternal investment is relatively low due to large body size (compared to the smaller species) and spread over longer periods of time [29]. Conversely, in most small primate species, reproductive success is linked primarily to food quality during the lush wet season when females give birth and lactate and infants are weaned (“income breeders”: Madagascar: [30]–[32]; New World: [33]). Nutrient availability during times of lactation and weaning would then represent a crucial factor for reproductive success [10], [30], [33], with adaptations to periods of food shortage potentially resulting in diversification [34]. Thus, once constraints imposed by seasonal fruit shortage had been solved through different adaptations [15], [35], primate communities could maintain more frugivorous taxa at sites where fruit protein concentrations were high enough to satisfy the protein needs of the lactating female and the infant during weaning. This would be relevant particularly in primate communities with a higher representation of small-bodied “income breeders”. Today, as well as in evolutionary times, primates of the Neotropics tend to be smaller than primates from Madagascar [6], [36]. Thus, the higher proportion of small frugivorous primate species in the Neotropics could have evolved if fruit protein content in the Neotropics would be above the primates' protein needs in the Neotropics, but below these requirements in Madagascar [24]. Based on this argument, we test the hypothesis that fruits in the Neotropics contain higher protein concentrations than fruits in Madagascar.

## Results

**Figure 1:**
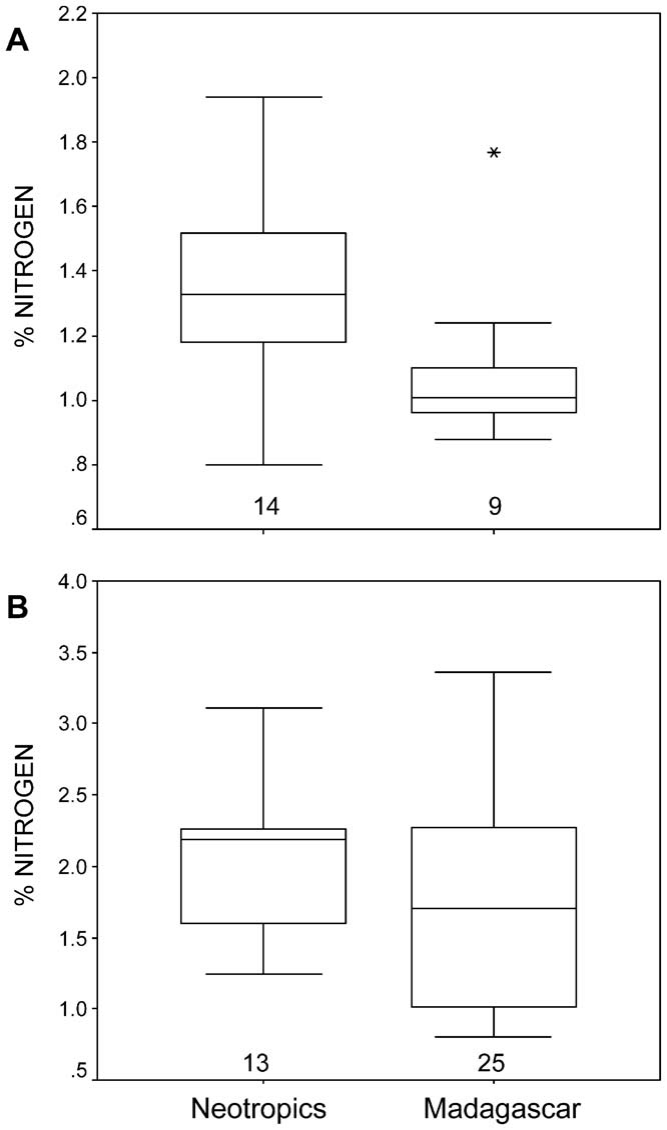
Average nitrogen concentration in fruits and primate vegetable foods in the Neotropics and in Madagascar. (A) Average nitrogen concentrations in fruits. Number of sites listed along the x-axis (Table 1). (B) Average nitrogen concentrations of all vegetable food items consumed by primates in the Neotropics and in Madagascar. Number of studies listed on the x-axis (Table 3).

The nitrogen concentrations in ripe fruits were significantly lower at sites in Madagascar than in the Neotropics (Madagascar: 1.09±0.28%, n = 9 sites; Neotropics: 1.37±0.29%, n = 14 sites; MWU-test: z = 2.49, p = 0.011; Fig. 1; Table 1).

**Table 1:** Nitrogen concentrations of fruits at different sites in Madagascar and the Neotropics.

Site	Country	% Nitrogen concentration in fruits (sample size)	Sampling	Source
**Madagascar**				
Anjamena (A)	Madagascar	0.88 (25)	PF: *Eulemur mongoz*	[57]
Kirindy/CFPF-CS7 (B)	Madagascar	1.10 (8)	PF: *Propithecus verreauxi*	Carrai unpubl.
Ranomafana (C)	Madagascar	0.96 (6)	PF: *Microcebus rufus*	[58]
Sahamalaza (1)	Madagascar	1.24±0.68 (67)	GS	Polowinsky & Schwitzer unpubl.
Kirindy/CFPF-N5 (2)	Madagascar	1.04±0.42 (40)	GS	[59], Bollen *et al*. unpubl.
Ranomafana (3)	Madagascar	1.01±0.40 (45)	PF: *Propithecus edwardsi*	Arrigo-Nelson unpubl.
Mandena (4)	Madagascar	0.99±0.81 (71)	GS	[60], Lahann unpubl.
Sainte Luce (5)	Madagascar	0.88± 0.39 (103)	GS	[61]
Berenty (6)	Madagascar	1.77±0.75 (11)	PF*: Microcebus griseorufus*	[62]
**Neotropics**				
Los Tuxtlas (D)	Mexico	1.34 (11)	PF: *Alouatta palliata*	[63]
Cockscomb Basin Wildlife Sanctuary (E)	Belize	1.33 (16)	PF: *Alouatta pigra*	[64]
Barro Colorado Island (F)	Panama	1.29 (8)	Several PF	[65]
Ilanos (G)	Venezuela	1.12 (9)	PF: *Alouatta seniculus*	[24]
Lago Guri (H)	Venezuela	1.47 (19)	PF: *Pithecia pithecia* + 3 fruits not eaten	[66]
Nouragues (I)	French Guiana	0.79 (14)	GS	[67]
San Cayetano (J)	Argentina	1.52 (2)	PF: *Alouatta caraya*	[68]
Mata Atlantica (K)	Brasil	1.18 (22)	PF: *Callicebus moloch*	[69]
Lomas Barbudal (11)	Costa Rica	1.33±0.75 (64)	PF: *Cebus capuchinus*	Vogel unpubl.
Tinigua National Park (12)	Columbia	1.28±0.77 (53)	PF: *Lagothrix lagotricha*	Stevenson unpubl.
Yasuní National Park (13)	Ecuador	1.59±0.79 (33)	PF + NPF: *Alouatta seniculus*	Derby unpubl.
Raleighvallen (14)	Suriname	1.17±0.54 (13)	PF: *Cebus apella*	Boinski & Vogel unpubl.
Parque Estadual Carlos Botelho (15)	Brasil	1.94±1.12 (4)	PF: *Brachyteles arachnoides*	Talebi unpubl.
Isla Brasilera (16)	Argentina	1.77 (1)	PF: *Alouatta caraya*	Kowalewski unpubl.

**Figure 2:**
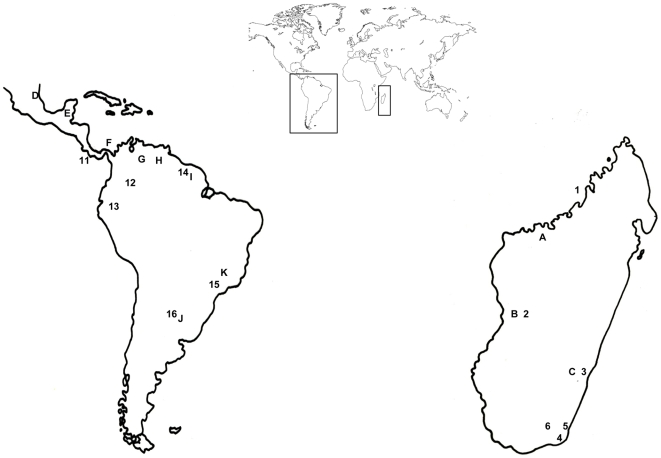
Sites for measures of fruit protein content (source of map: www.smithLifeScience.com/Tools.htm; free). Site labels are listed in Table 1. Letters refer to studies published previously. Numbers refer to new and yet unpublished studies.

Letters and numbers listed in the first column refer to sites shown in Figure 2. Values of the published datasets (sites marked by letters in the left hand column and in Figure 2) are means or medians as listed in the original reference; sample size listed in brackets. For samples analyzed in the context of the present paper (sites marked by numbers) values are means and standard deviations. Nitrogen concentrations were recorded by general sampling (GS) of fruits from woody plant species available during the study period, or of fruits consumed by a specific primate species (PF) plus fruits not consumed by primates at the sites (NPF).

Sites are represented with different sample size. If the analyses were based on the individual plant species analyzed at each site, fruits from Madagascar contained 1.04±0.60% nitrogen (n = 334) while New World fruits contained on average 1.37±0.73% nitrogen (n = 168; MWU test: z = 5.08, p<0.001). Since the raw data are not available for most of the published studies, this analysis was restricted to the unpublished data marked with numbers in Table 1 and Figure 2 (six sites in the Neotropics and six sites in Madagascar).

Given differences in fig availability between Madagascar and the Neotropics, it appeared possible that figs might play different roles in Madagascar and in the Neotropics. However, when figs were removed from the analyses, the two regions still differed significantly with average nitrogen contents of 1.04±0.61% in Madagascar (n = 322 samples) and of 1.40±0.76% in the Neotropics (n = 157 samples; MWU test: z = 5.19, p<0.001).

In contrast to the nitrogen concentrations of fruits, the nitrogen concentrations in the overall vegetable diet of primates (including leaves, exudates, flowers, fruits) did not differ between species of the Neotropis and of Madagascar (MWU-test: z = 1.28, p>0.05; Neotropics: mean nitrogen content of vegetable primate food: 2.05±0.57, n = 13 studies at 13 different sites and 9 different primate species; Madagascar: 1.74±0.73, n = 25 studies at 11 different sites and 23 different primate species; Fig. 1; Table 2).

**Table 2:** Mean nitrogen concentration of all vegetable food items consumed by various primate species in Madagascar and the Neotropics.

Species	Site	Country	N	% Nitrogen	Source
**Madagascar**					
*Avahi laniger*	Ranomafana	Madagascar	5	2.5	[70]
*Avahi meridionalis*	Sainte Luce	Madagascar	39	1.2	Norscia unpubl.
*Cheirogaleus major*	Mandena	Madagascar	77	0.9	[60]
*Cheirogaleus medius*	Sainte Luce	Madagascar	33	0.8	[59]
*Cheirogaleus medius*	Mandena	Madagascar	75	0.9	[60]
*Eulemur collaris*	Sainte Luce	Madagascar	100	1.0	Donati unpubl.
*Eulemur macaco*	Ampasikely	Madagascar	23	1.7	[71]
*Eulemur flavifrons*	Sahamalaza	Madagascar	88	1.6	Polowinsky & Schwitzer unpubl.
*Eulemur mongoz*	Anjamena	Madagascar	46	1.1	[57]
*Eulemur rufus*	Kirindy	Madagascar	20	1.0	[59]
*Hapalemur alaotrensis*	Alaotra	Madagascar	15	2.1	[72]
*Hapalemur aureus*	Ranomafana	Madagascar	63	3.2	Tan unpubl.
*Hapalemur griseus*	Ranomafana	Madagascar	40	3.4	Tan unpubl.
*Hapalemur merdidionalis*	Mandena	Madagascar	26	1.6	Ralison unpubl.
*Hapalemur simus*	Ranomafana	Madagascar	141	2.3	Tan unpubl.
*Indri indri*	Mantadia	Madagascar	10	1.7	[73]
*Lemur catta*	Berenty	Madagascar	28	2.4	[74]
*Lepilemur ruficaudatus*	Kirindy N5	Madagascar	194	2.4	[26], [75]
*Microcebus griseorufus*	Berenty	Madagascar	25	1.5	[62]
*Microcebus murinus*	Mandena	Madagascar	77	0.9	[60]
*Microcebus rufus*	Ranomafana	Madagascar	12	0.9	[76]
*Propithecus diadema*	Mantadia	Madagascar	10	2.0	[73]
*Propithecus edwardsi*	Ranomafana	Madagascar	392	2.0	Arrigo-Nelson unpubl.
*Propithecus verreauxi*	Kirindy CS7	Madagascar	246	2.2	Carrai unpubl.
*Propithecus verreauxi*	Kirindy N5	Madagascar	14	2.3	Ganzhorn unpubl.
**Neotropics**					
*Alouatta caraya*	San Cayetano	Argentina	16	2.2	[68]
*Alouatta caraya*	Isla Brasilera	Argentina	30	2.8	Kowalewski unpubl.
*Alouatta palliata*	Barro Colorado Island	Panama	5	2.0	[65]
*Alouatta palliata*	Los Tuxtlas	Mexico	71	2.2	[63], [77]
*Alouatta pigra*	Community Baboon Sancuary and Cockscomb Basin Wildlife Sanctuary	Belize	124	3.1	[64]
*Alouatta seniculus*	Yasuní NP	Ecuador	124	2.3	Derby unpubl.
*Alouatta seniculus*	Ilanos	Venezuela	37	2.4	[24]
*Ateles geoffroyi*	Barro Colorado Island	Panama	4	1.9	[65]
*Brachyteles arachnoides*	Parque Estadual Carlos Botelho	Brasil	10	2.2	Talebi unpubl.
*Callicebus moloch*	Mata Atlantica	Brasil	32	1.6	[69]
*Cebus capuchinus*	Barro Colorado Island	Panama	3	1.2	[65]
*Cebus capuchinus*	Barbudal	Costa Rica	65	1.4	Vogel unpubl.
*Lagothrix lagothricha*	Tinigua National Park	Columbia	53	1.3	[78]

## Discussion

On average, protein concentrations in the vegetable food of primates do not differ between regions. This indicates, that the protein requirements of species from different radiations are independent of their phylogenetic history, even though some of the strepsirhine primates of Madagascar can have reduced metabolic rates [37], [38]. In contrast to the overall vegetable food composition, fruits in the Neotropics and in Madagascar vary in their nitrogen concentrations. This pattern is not due to different sample size, since, despite the fact that fruits have been sampled most comprehensively in Madagascar, the larger number of samples per site does not lower the average protein concentration when compared to the fruits eaten most frequently (Table 3). Thus, the differences between regions are unlikely due to sampling artifacts.

**Table 3:** Fruit selection of primates in relation to nitrogen concentrations; ns = not significant.

Species	Site	Country	% Nitrogen in fruits eaten (sample size)	% Nitrogen in fruits not eaten (sample size)	Relationship between the frequency of consumption and nitrogen concentration	Source
**Madagascar**						
*Microcebus murinus*	Mandena	Madagascar	0.9 (61)	0.8 (14)	ns	[60]
*Cheirogaleus medius*	Mandena	Madagascar	0.9 (59)	0.8 (16)	ns	[60]
*C. medius*	Kirindy/CFPF	Madagascar	0.9 (23)	1.0 (18)	ns	[59]
*C.* spp.	Sainte Luce	Madagascar	0.8 (33)	0.8 (64)	ns	[59]
*C. major*	Mandena	Madagascar	0.9 (63)	0.8 (14)	ns	[60]
*Eulemur collaris*	Sainte Luce	Madagascar	0.9 (86)	0.8 (18)	ns	[59], [79]
*E. rufus*	Kirindy/CFPF	Madagascar	1.0 (20)	0.9 (25)	ns	[59]
**Neotropics**						
*Lagothrix lagotricha*	Tinigua National Park	Columbia			ns	[78], Stevenson, unpubl.
*Cebus apella*	Manu National Park	Brazil			Negative correlation between frequency of consumption and nitrogen concentration	[80]
*Callicebus moloch*	Mata Atlantica	Brazil	1.2 (22)		ns	[69]

The protein requirements of primates are such that foods consumed should contain about 7–11% protein (equivalent to 1.1–1.8% nitrogen). These values include the consumption of leaves with secondary components inhibiting digestion [24], [39]. Even cattle and sheep with improved nitrogen digestion due to rumination avoid food with nitrogen contents below 1.1% [40]. Thus, fruits at sites in Madagascar with an average nitrogen concentration of 1.0–1.1% are on the lower end of nitrogen concentrations found in sampled fruits of the Neotropics and are below the nitrogen requirements for primates. In conjunction with environmental conditions which seem less predictable in Madagascar than in other parts of the world [11], [12], this might have contributed to the evolution of very few frugivorous and the high proportion of folivorous lemur species in Madagascar. Similarly, this might also explain the low representation of frugivorous bird species on Madagascar [1], [41].

The contemporary low representation of frugivorous lemur species is not an artifact of recent extinctions. Madagascar has lost its large vertebrate species (elephant birds, giant tortoises, pygmy hippopotamus, large lemurs) during the last millennium [42]. But except for two species of *Pachylemur*, none of these species seem to have relied on fruits as a staple diet [43].

In contrast to the situation in Madagascar, the average nitrogen concentrations of fruits in the New World are well within the nitrogen requirements of primates. Thus, the selection pressure to extend their diet beyond fruits seems to be lower in the Americas than it is in Madagascar.

The difference in nitrogen concentrations between regions may not appear to be biologically substantial (i.e.: 1.0–1.1% nitrogen in Madagascar and 1.4% in the Neotropics). Yet, assuming that animal-dispersed fruits evolved under the evolutionary pressure to attract dispersers without investing too much, the difference in nitrogen of around 0.3% between Madagascar and the Neotropics is likely to be quite relevant; from the plants' perspective, this small increment represents an increase in nitrogen investment (and nitrogen loss once the fruits are eaten) of about 30% between Madagascar and the New World fruits. Given that many trees are exhausted especially after mast fruiting [44], a 30% difference in the protein investments in fruits - and in a major food resource - must have profound consequences on the plant as well as on the consumer communities.

The hypothesis presented here should be considered as one of several constraining factors of evolutionary relevance. It is based on food quality and does neither consider quantitative aspects, nor does it take into account the need to match the animals' energy requirements (e.g., [45], [46]), specific mineral needs [47], or avoidance of plant secondary components [48]. However, these possibly confounding variables can not be separated as long as we do not have the means to measure the qualitative and quantitative availability of food, its individual and seasonal variation as well as seasonal or ontogenetic variation in ingestion and nutrient assimilation by the animals (e.g., [49], [50]).

## Materials and Methods

### Database

We compiled data on the nitrogen concentrations of ripe fruits in forests of Madagascar and in the New World from the literature and supplemented the data with additional analyses of fruits from six sites in the Neotropics and six sites in Madagascar. Non-forest habitats were not considered. We used nitrogen concentrations as measured by the Kjeldahl procedure rather than crude protein in this comparison, because different conversion factors from nitrogen to crude protein have been suggested [51]–[53]. As other measures of protein concentrations, such as ninhydrin, Biorad, or amino acids can not be transformed to nitrogen concentrations using a simple transformation factor, only studies reporting total nitrogen measured with the Kjeldahl method were used in the present analysis. Few ripe fruits contain digestion inhibitors such as frequently found in leaves. Thus, there was no need to control for these digestion-inhibiting components [54], [20]. Except for the samples collected by Polowinsky and Schwitzer (unpubl.), all other samples collected by the authors for the present paper (listed in Table 1) were analyzed with the same equipment and procedure in the labs of JUG [55].

Community-wide data on the chemical composition of fruits are scant. However, of 10 published primatological studies addressing protein selection in fruits in Madagascar and the Neotropics (and several others from Africa, not considered here; plus several unpublished studies from Madagascar and the Neotropics), none found a positive significant difference in nitrogen concentrations between those fruits eaten and not eaten by the primates under study. One study that did report a significant difference, reported a *negative* correlation between consumption and protein concentrations (Table 3). Therefore, we consider the fruits consumed by primates as a conservative representative sample of the nitrogen concentrations for all fruits available at each site. Thus, we use three types of data to characterize fruit nitrogen content of a given site: (1) comprehensive sampling of all fruits obtained during a study, (2) fruits eaten by primates, and (3) fruits eaten and not eaten by primates if non-eaten fruits had been collected for comparisons (Table 1).

To control for possible physiological differences between primate radiations (such as between the lemurs of Madagascar and haplorhine primates in the Neotropics), we investigated whether the nitrogen content of all dietary plant components (fruits, leaves, flowers, seeds) differed between primate radiations. According to our hypothesis, the diet of the species of the different primate radiations should not differ in the nitrogen concentrations of their overall diet, but fruits in the New World should have higher nitrogen concentrations than in Madagascar.

### Statistical Analysis

Since data deviated from normality, we applied non-parametric tests for the comparisons. Tests were run with SPSS 9.0 [56].
